# Insulin Therapy Is Associated With an Increased Risk of Carotid Plaque in Type 2 Diabetes: A Real-World Study

**DOI:** 10.3389/fcvm.2021.599545

**Published:** 2021-02-01

**Authors:** Jiang-Feng Ke, Jun-Wei Wang, Zhi-Hui Zhang, Ming-Yun Chen, Jun-Xi Lu, Lian-Xi Li

**Affiliations:** Shanghai Key Laboratory of Diabetes Mellitus, Department of Endocrinology and Metabolism, Shanghai Clinical Center for Diabetes, Shanghai Key Clinical Center for Metabolic Diseases, Shanghai Diabetes Institute, Shanghai Jiao Tong University Affiliated Sixth People's Hospital, Shanghai, China

**Keywords:** insulin therapy, type 2 diabetes, atherosclerosis, carotid plaque, insulin resistance

## Abstract

**Background:** Controversies concerning the association between insulin therapy and atherosclerotic lesions in type 2 diabetes mellitus (T2DM) remain to exist. The purpose of this study was to investigate whether insulin therapy in T2DM patients is linked with the increased risk of carotid atherosclerosis in real-world settings.

**Methods:** We retrospectively enrolled 2,356 hospitalized patients with T2DM, including 1,716 subjects receiving insulin therapy and 640 subjects without receiving insulin therapy. Carotid atherosclerotic lesions including carotid intima-media thickness (CIMT), carotid plaque and carotid stenosis were assessed by Doppler ultrasonography and were compared between T2DM patients treated with and without insulin.

**Results:** After adjusting for age and duration of diabetes, there was a significant increase in the prevalence of carotid plaque in both men (52.0 vs. 41.7%, *p* = 0.007) and women (49.6 vs. 39.7%, *p* = 0.003) receiving insulin therapy than in those without receiving insulin therapy. After further controlling for other confounding factors, compared with the patients without receiving insulin therapy, the risk of carotid plaque was still significantly increased not only in women treated with insulin (OR: 1.810; 95% CI: 1.155–2.837, *p* = 0.010), but also in men treated with insulin (OR: 1.867; 95% CI: 1.307–2.666; *p* = 0.001). Additionally, HOMA2-B% was higher in both women and men without receiving insulin therapy compared with those receiving insulin therapy (*p* < 0.001 in both men and women), but HOMA-IR was significantly higher in patients treated with insulin than in those without receiving insulin therapy (*p* < 0.001 in both men and women).

**Conclusions:** Insulin therapy is associated with markedly increased risk of carotid atherosclerotic lesions in type 2 diabetes, which partly attribute to the more serious insulin resistance in T2DM patients receiving insulin therapy.

## Introduction

Insulin therapy is one of the most common methods to control blood glucose in patients with Type 2 diabetes mellitus (T2DM). A number of studies found that insulin therapy was associated with the decreased risk of atherosclerotic lesions in type 2 diabetes, which partly attribute to the glucose-lowering effect of insulin (1–3). For instance, the 10-year post-trial follow-up of UKPDS analyzed the association between intensive glucose therapy and atherosclerotic outcomes in T2DM (1). After long-term clinical follow-up, a close association was observed between sulfonylurea–insulin therapy and decreased risk of cardiovascular morbidity and mortality in T2DM patients (1). Additionally, the study also found a reduced risk of myocardial infarction and death from any cause in patients receiving sulfonylurea–insulin therapy (1). Furthermore, a retrospective cohort analysis showed that 19 patients/1,000 patient-years suffered a cardiovascular event in subjects receiving insulin therapy compared with 22/1,000 patient-years in subjects receiving non-insulin treatment, which indicates that insulin treatment may be related to reduced risk of atherosclerotic outcomes in type 2 diabetes (3).

However, recent studies have thrown doubt on the benefits of tight glycemic control, especially using insulin, on atherosclerotic lesions in subjects with long-standing T2DM (4–8). For example, two recent studies demonstrated that intensive glucose control failed to improve survival and outcomes of macrovascular complications in the first 10 years in patients with diabetes, and that insulin-related morbidity and mortality increased with higher insulin doses and decreased with concomitant metformin treatment (4, 7). Furthermore, another study found that after multivariable adjustment, T2DM subjects treated with insulin suffered a higher risk of all-cause mortality though bias cannot be excluded, as insulin was more likely to be used in subjects with more severe diabetes (8). Also, the Euro Heart Survey on Diabetes and the Heart investigated the impact of different glucose-lowering modalities on cardiovascular events including death, myocardial infarction and stroke, and found that insulin treatment may associated with a more serious prognosis in DM patients with coronary artery disease (9).

Therefore, the association between insulin treatment and atherosclerosis and its complications in T2DM has not been definitively determined. The purpose of this study was to investigate the association between insulin therapy and carotid atherosclerotic lesions, including CIMT, carotid plaque and stenosis in real-world settings in Chinese subjects with type 2 diabetes.

## Methods

### Study Population and Design

Between January 2007 and June 2009, 3,598 patients with type 2 diabetes who were hospitalized in our department were consecutively observed. The inclusion criteria were as follows: type 2 diabetes diagnosed according to WHO criteria (10); age ≥ 17 years old; carotid ultrasound examination performed; subjects treated with or without insulin; insulin treatment more than 6 months if receiving insulin therapy. All participants' information was recorded, such as the history of hypertension, duration of diabetes (DD), smoking and alcohol use. The definition of hypertension, smoking, and alcohol status has been described in our previous studies (11–13). In detail, smoking included both current and former smokers. Likewise, alcohol use included current and former use of alcohol.

Insulin therapy was defined as continuous use of insulin for at least 6 months. Based on the inclusion criteria, 1,242 subjects were eliminated, and ultimately, 2,356 patients were included in the final analyses. This real-world study was approved by the ethics committee of Shanghai Jiao Tong University Affiliated Sixth People's Hospital. Written informed consent was obtained from all patients.

### Physical Examination and Laboratory Measurements

Physical examinations including height, weight, waist circumference, hip circumference, and blood pressure were performed according to standard protocols as previously described (11–13). Both body mass index (BMI) and waist-to-hip ratio (WHR) were calculated using the corresponding formula (14).

After admission to the hospital, patients continued to be given insulin with different regimens of insulin therapy if they treated with insulin before admission. The laboratory measurements have been described in detail in our previous study (11, 12). That is, venous blood samples were drawn after an overnight fast and 2 h after breakfast on the second day after admission. In detail, serum insulin and C-peptide concentration were determined by Elecsys (Roche, LOT 43022303, LOT45290601, respectively) on an analyzer (Roche, cobas e 601, Shanghai, China).

In brief, the laboratory evaluations included fasting plasma glucose (FPG), 2-h postprandial plasma glucose (2h PPG), fasting plasma insulin (Fins), 2-h postprandial plasma insulin (2h Pins), fasting C-peptide (FCP), 2-h postprandial C-peptide (2h PCP), glycosylated hemoglobin A1C (HbA1C), blood platelet count (BPC), white blood cell count (WBCC), and C-reactive protein (CRP), alanine aminotransferase (ALT), aspartate aminotransferase (AST), total triglycerides (TTG), total cholesterol (TC), high-density lipoprotein cholesterol (HDL-C), low-density lipoprotein cholesterol (LDL-C), creatinine (Cr), serum uric acid (SUA), and urinary albumin excretion (UAE). The 24 h UAE was determined as the mean of the values obtained from three separate early morning urine samples during the period of hospitalization. The estimated glomerular filtration rate (eGFR) was calculated by equation for Chinese individuals: eGFR = 175 × (serum creatinine) – 1.234 × (age) – 0.179 (×0.79 if female) (15). Insulin resistance (HOMA-IR) and β-cell function (HOMA2-B%) were calculated using the corresponding formula (16, 17).

### Ultrasonographic Examination and Diagnostic Criteria

Doppler ultrasonography examinations of carotid arteries, including the measurement of carotid intima-media thickness (CIMT), atherosclerotic plaque and stenosis, had been described in details in our previous study (11, 12, 18). That is, three experienced sonographers performed ultrasound examinations using an Acuson Sequoia 512 machine with a probe frequency of 5–13-MHz, according to standardized protocols (11, 12, 18). The definitions of CIMT, carotid plaque and stenosis had also been described in details in our previous studies (11, 12, 14, 18).

### Statistical Analysis

Statistical tests were performed using the SPSS 15.0 (SPSS Inc., Chicago, IL, USA). A value of *P* < 0.05 was considered to indicate a statistically significant difference. Data were expressed as mean ± SD, percentages or medians (interquartile range 25–75%) for skewed variables. One-way ANOVA with LSD and independent sample *t*-tests was applied to compare normally distributed continuous variables. The Mann–Whitney U test and Kruskal–Wallis H test were used to analyze continuous variables not distributed normally. The prevalence of data was analyzed by χ^2^ test. Both binary logistic and general linear regressions were applied to compare differences in the variables while controlling for other factors. Five models were constructed to assess the associations of the carotid plaque with insulin therapy by logistic regression: model 1 included adjustments for age, DD, hypertension, smoking status, and alcohol use; model 2 included further adjustments for use of anti-hypertensive agents (AHAs), lipid-lowering drugs (LLDs), anti-platelet agents (APAs) and metformin; model 3 included further adjustment for SBP, DBP, BMI, WC, and WHR; model 4 included additional adjustments for WBCC, BPC, ALT, AST, TG, TC, HDL-C, LDL-C, Cr, SUA, UAE, eGFR, CRP, FPG, 2h PPG, HbA1C, FCP, 2h PCP; and model 5 included additional adjustments for Fins and 2h Pins.

## Results

### Clinical Characteristics of the Subjects

Table 1 presents the clinical and laboratory characteristics of the patients treated with and without insulin. There was no significant difference of age and gender between the subjects treated with and without insulin. Compared with the subjects without receiving insulin therapy, the subjects receiving insulin therapy had longer DD, higher SBP, WBCC, CRP, HbA1C, TC, LDL-C, FPG, 2h PPG, Fins, 2h Pins, and 24h UAE levels and had lower BMI, FCP, 2h PCP, TG, and SUA (all *p* < 0.05).

**Table 1:** Characteristics of the studied subjects.

**Variables**	**Subjects without insulin therapy (*n* = 640)**	**Subjects with insulin therapy (*n* = 1,716)**	***p* value**
Male (*n*, %)	348 (54.4%)	917 (53.4%)	0.685
Age (years)	60 ± 11	61 ± 12	0.168
^*^DD (months)	48 (12–108)	96 (24–156)	<0.001
Smoking (*n*, %)	167 (26.1%)	465 (27.1%)	0.625
Alcohol (*n*, %)	92 (14.4%)	243 (14.2%)	0.895
Hypertension (*n*, %)	355 (55.5%)	957 (55.8%)	0.896
LLD (*n*, %)	196 (30.6%)	531 (30.9%)	0.881
AHAs (*n*, %)	313 (48.9%)	886 (51.6%)	0.239
APAs (*n*, %)	332 (51.9%)	928 (54.1%)	0.340
Metformin (*n*, %)	405 (63.3%)	1,029 (60.0%)	0.142
SBP (mmHg)	131 ± 16	134 ± 18	0.001
DBP (mmHg)	80 ± 9	80 ± 10	0.605
BMI (kg/m^2^)	25.1 ± 3.3	24.8 ± 3.5	0.039
WC (cm)	89.4 ± 10.2	89.4 ± 10.5	0.869
WHR	0.9 ± 0.1	0.9 ± 0.1	0.086
^*^WBCC (10^9^/L)	5.9 (5.0–6.8)	6.2 (5.2–7.4)	<0.001
^*^BPC (10^12^/L)	180 (153–214)	183 (151–224)	0.256
^*^FPG (mmol/l)	6.72 (5.77–8.15)	8.12 (6.50–10.10)	<0.001
^*^2h PPG (mmol/l)	11.51 (9.06–14.12)	14.30 (10.57–17.55)	<0.001
HbA1C (%)	7.59 ± 1.68	9.38 ± 2.18	<0.001
^*^FCP (ng/mL)	2.15 (1.65–2.95)	1.40 (0.84–2.10)	<0.001
^*^2h C-P (ng/mL)	5.29 (4.06–5.97)	2.90 (1.64–4.59)	<0.001
^*^Fins (mU/L)	12.56 (9.30–17.94)	16.98 (11.47–26.58)	<0.001
^*^2h Pins (mU/L)	46.28 (30.71–70.82)	62.85 (42.19–90.21)	<0.001
^*^TG (mmol/l)	1.52 (1.03–2.12)	1.38 (0.96–2.1)	0.003
TC (mmol/l)	4.56 ± 0.93	4.73 ± 1.17	<0.001
HDL-C (mmol/l)	1.10 ± 0.27	1.13 ± 0.32	0.065
LDL-C (mmol/l)	3.01 ± 0.81	3.11 ± 0.98	0.008
^*^ALT (U/l)	19 (13–29)	18 (13–28)	0.225
^*^AST (U/l)	19 (16–24)	18 (15–24)	0.127
^*^Cr (μmol/l)	68 (57–79)	66 (55–82)	0.778
^*^SUA (μmol/l)	324 (270–378)	305 (253–373)	<0.001
^*^UAE (mg/24 h)	9.34 (5.83–18.05)	13.05 (7.03–47.34)	<0.001
^*^eGFR (ml/min/1.73) m^2^)	106.29 (91.41–125.40)	108.33 (86.32–133.26)	0.682
^*^CRP (mg/l)	0.98 (0.44–2.10)	1.17 (0.50–3.32)	<0.001

*Values are expressed as the mean ± S.D, or median with interquartile range, or percentages*.

* *The Mann-Whitney U–test was applied*.

*P-value: The p-values were not adjusted for age and sex for the trend*.

### Percentage of the Subjects Treated With Insulin Stratified by Sex, Age, and DD

**Figure 1:**
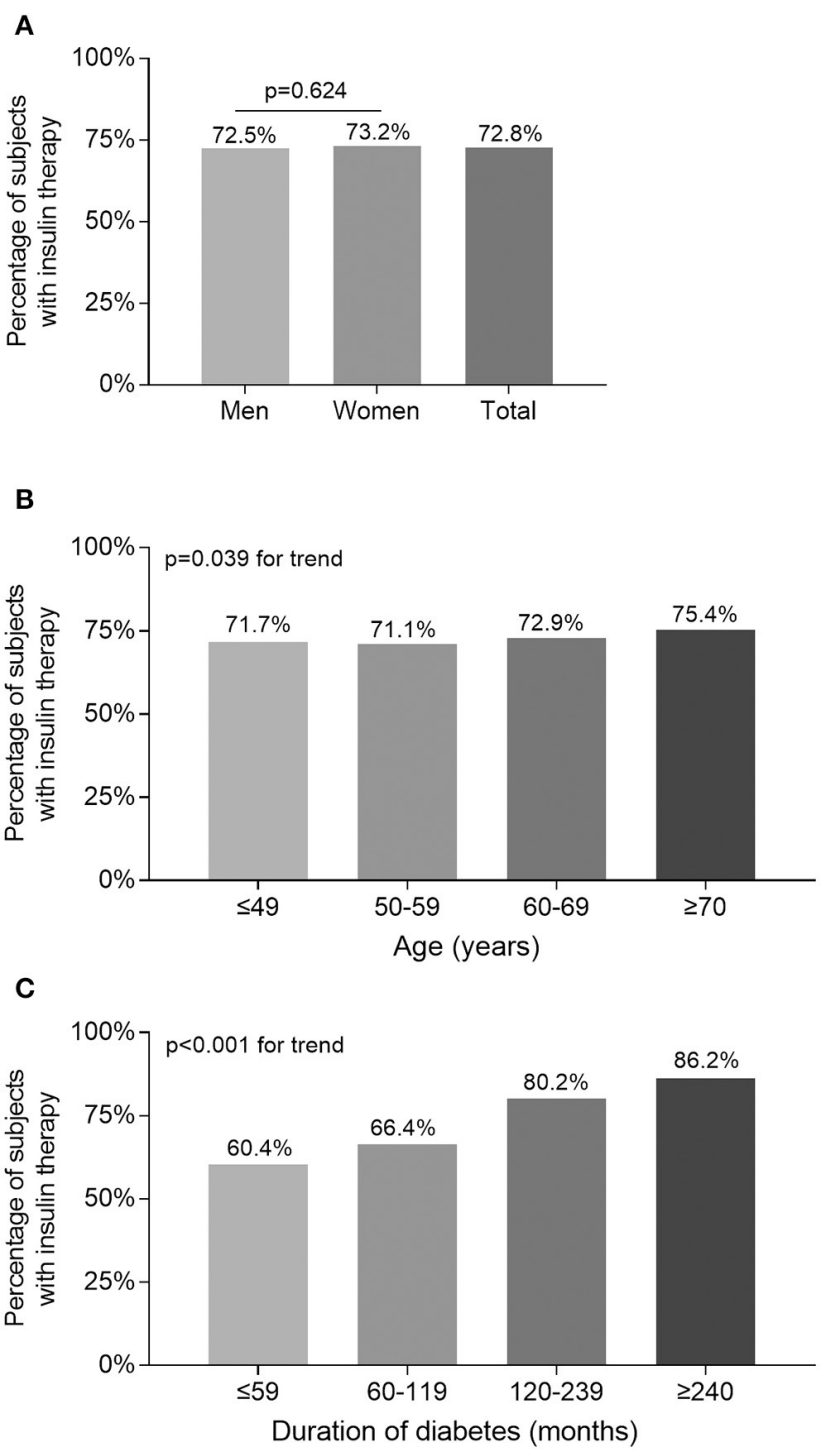
Comparison of the percentage of the subjects receiving insulin therapy stratified by sex, age, and DD. **(A)** Comparison of the percentage of the subjects receiving insulin therapy stratified by sex after adjusting for age and DD. **(B)** Comparison of the percentage of the subjects receiving insulin therapy stratified by age after adjusting for sex and DD. **(C)** Comparison of the percentage of the subjects receiving insulin therapy stratified by DD after adjusting for sex and age.

Percentage of the subjects treated with insulin stratified by sex, age, and DD are presented in Figure 1. After controlling for age and DD, there was no sex-related significant difference in the percentage of the subjects receiving insulin therapy (Figure 1A). However, there were significantly increased trend in the percentage of the subjects receiving insulin therapy with increased age (*p* = 0.039 for trend) and prolonged duration of diabetes (*p* < 0.001 for trend) (Figures 1B,C).

### Comparison of Carotid Atherosclerotic Lesions Between the Patients Treated With and Without Insulin

**Figure 2:**
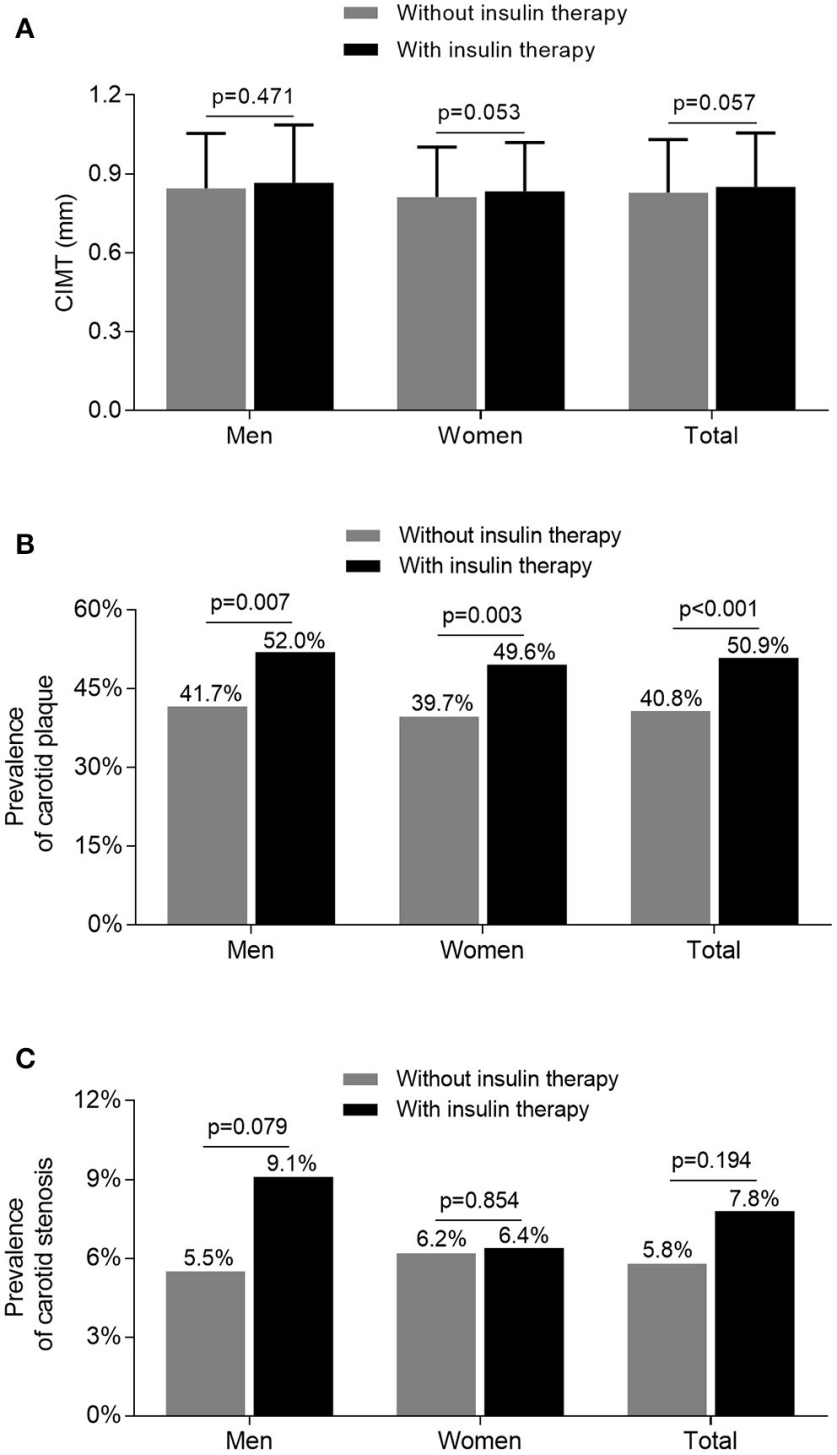
Comparison of carotid atherosclerotic lesions stratified by sex between the patients treated with and without insulin. **(A)** Comparison of the value of CIMT stratified by sex between the patients treated with and without insulin after adjusting for age and DD. **(B)** Comparison of the prevalence of carotid plaque stratified by sex between the patients treated with and without insulin after adjusting for age and DD. **(C)** Comparison of the prevalence of carotid stenosis stratified by sex between the patients treated with and without insulin after adjusting for age and DD.

Figure 2 illustrates the comparison of carotid atherosclerotic lesions between the subjects treated with and without insulin. After adjusting for age and DD, the prevalence of carotid plaque was significantly higher in the subjects receiving insulin therapy than in those without receiving insulin therapy (52.0 vs. 41.7%, *p* = 0.007 for men; 49.6 vs. 39.7%, *p* = 0.003 for women) (Figure 2B). However, although the value of CIMT and prevalence of carotid stenosis were higher in the subjects receiving insulin therapy compared with the subjects without receiving insulin therapy, the difference was not significant after controlling for age and DD (Figures 2A,C).

### Comparison of Insulin Resistance and β-Cell Function Between the Patients Treated With and Without Insulin

**Figure 3:**
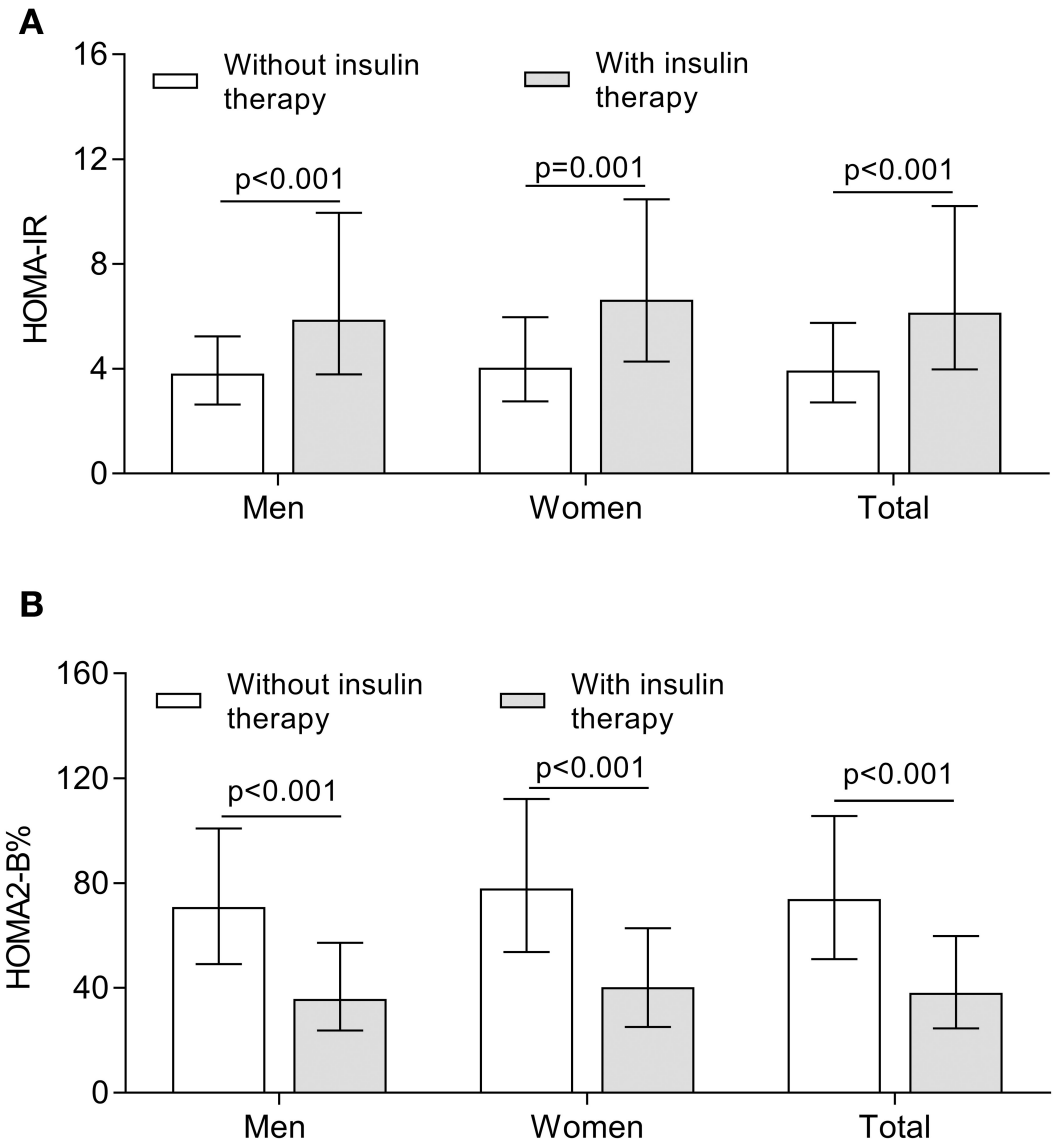
Comparison of insulin resistance and β-cell function between the patients treated with and without insulin. **(A)** Comparison of the value of HOMA-IR stratified by sex between the patients treated with and without insulin after controlling for age and DD. **(B)** Comparison of the value of HOMA2-B% stratified by sex between the patients treated with and without insulin after controlling for age and DD.

Figure 3 demonstrates the comparison of insulin resistance and β-cell function between the subjects treated with and without insulin. After controlling for age and DD, the higher value of HOMA-IR calculated by Fins was observed in both women and men receiving insulin therapy in comparison with the subjects without receiving insulin therapy (all *p* < 0.001) (Figure 3A). Interestingly, the HOMA2-B% value calculated by FCP was significantly higher in the subjects without receiving insulin therapy compared with the subjects receiving insulin therapy (all *p* < 0.001) (Figure 3B).

### Association of Insulin Therapy With Carotid Plaque

Table 2 displays the association between insulin therapy and carotid plaque in type 2 diabetes. After adjustment for age, DD, hypertension, smoking status, and alcohol use (Model I), insulin therapy was independently associated with the presence of carotid plaque in both women (OR: 1.619, 95% CI: 1.200–2.185, *p* = 0.002) and men (OR: 1.461, 95% CI: 1.103–1.935, *p* = 0.008) with type 2 diabetes. After additional controlling for use of AHAs, LLDs, APAs and metformin (Model 2), insulin therapy still retained an independent association with carotid plaque in both women (OR: 1.591, 95% CI: 1.160–2.183, *p* = 0.004) and men (OR: 1.553, 95% CI: 1.155–2.089, *p* = 0.004). Moreover, after further adjustment for SBP, DBP, BMI, WC and WHR (Model 3) and for WBCC, BPC, ALT, AST, TG, TC, HDL-C, LDL-C, Cr, SUA, UAE, eGFR, CRP, FPG, 2h PPG, HbA1C, FCP, 2h PCP, (Model 4), insulin therapy was still significantly associated with the presence of carotid plaque in both women (Model 3: OR: 1.535, 95% CI: 1.104–2.133, *p* = 0.011; Model 4: OR: 1.709, 95% CI: 1.179–2.478, *p* = 0.005) and men (Model 3: OR: 1.541, 95% CI: 1.132–2.097, *p* = 0.006; Model 4: OR: 1.760, 95% CI: 1.234–2.508, *p* = 0.002) with type 2 diabetes. Intriguingly, after further adjustment for Fins and 2h Pins (Model 5), significant association was still observed between insulin therapy and the presence of carotid plaque in both women (Model 5: OR: 1.810, 95% CI: 1.155–2.837, *p* =0.010) and men (Model 5: OR: 1.867, 95% CI: 1.307–2.666, *p* = 0.001) with type 2 diabetes.

**Table 2:** Association between carotid plaque and insulin therapy.

	**Men**		**Women**		**All**	
	**OR (95% CI)**	***p* values**	**OR (95% CI)**	***p* values**	**OR (95% CI)**	***p* values**
Model 1	1.461 (1.103–1.935)	0.008	1.619 (1.200–2.185)	0.002	1.525 (1.243–1.871)	<0.001
Model 2	1.553 (1.155–2.089)	0.004	1.591 (1.160–2.183)	0.004	1.557 (1.255–1.932)	<0.001
Model 3	1.541 (1.132–2.097)	0.006	1.535 (1.104–2.133)	0.011	1.508 (1.205–1.889)	0.001
Model 4	1.760 (1.234–2.508)	0.002	1.709 (1.179–2.478)	0.005	1.633 (1.237–2.156)	0.001
Model 5	1.867 (1.307–2.666)	0.001	1.810 (1.155–2.837)	0.010	1.695 (1.131–2.196)	<0.001

*Model 1: Adjusted for age, DD, hypertension, smoking status, and alcohol use (gender adjustment for all subjects)*.

*Model 2: Further adjustment for use of AHAs, LLDs, APAs, and metformin (gender adjustment for all subjects)*.

*Model 3: Further adjustment for SBP, DBP, BMI, WC, and WHR (gender adjustment for all subjects)*.

*Model 4: Further adjustment for WBC, BPC, ALT, AST, TG, TC, HDL-C, LDL-C, Cr, SUA, UAE, eGFR, CRP, FPG, 2h PPG, HbA1C, FCP, 2h PCP*.

*Model 5: Further adjustment for Fins and 2h Pins*.

## Discussion

The association between insulin therapy and atherosclerotic lesions in T2DM subjects has not been clearly confirmed so far. Therefore, we performed this real-world study to examine whether insulin therapy is related to increased risk of carotid atherosclerotic lesions in T2DM. In fact, we observed a strongly positive association between insulin therapy and the presence of carotid plaque in T2DM, even after adjusting for traditional cardiovascular risk factors. To the best of our knowledge, this is the first time to study the association between insulin therapy and carotid atherosclerotic lesions in Chinese patients with type 2 diabetes.

The percentage of the T2DM patients receiving insulin therapy was 72.5% in men, and 73.2% in women, which was higher than the results from the study by Kornowski et al., who found the percentage of insulin therapy was 33.3% in men, and 40.4% in women (19). Consistent with other studies, we also found the percentage of the subjects receiving insulin therapy in T2DM patients successively increased with increasing age and prolonging DD, which presumably resulted from gradual deterioration of insulin secretion with the increase of age and the extension of diabetes duration (20, 21). However, there was no sex-related significant difference in the percentage of subjects receiving insulin therapy in our study, which is in keeping with another study performed by Kalkman et al. (22).

So far, although a number of studies have investigated the association between insulin treatment and atherosclerosis and its complications, the relationship between them have not been well established and the controversies remain to exist (22, 23). Some investigations showed a beneficial effect of insulin treatment on diabetic atherosclerosis and macrovascular complications in type 2 diabetes. For example, an early study found that thepatients treated with insulin ≥ 1 year had less reference plaque and stenosis plaque, and smaller reference arterial areas and stenosis arterial areas compared with those without receiving insulin treatment, which may attribute to impaired adaptive remodeling and arterial shrinkage (22). In addition, Malmberg et al. found that the patients receiving multiple daily insulin treatment during acute myocardial infarction had 11% of reduction in mortality compared with those treated with conventional medications after at least 3.4 years of follow-up (23, 24). Furthermore, another study showed that intensified insulin-based glycemic control had a long-term impact on life expectancy, with an average survival increase of 2.3 years and lasting at least 8 years in DM patients with acute myocardial infarction (23). Therefore, insulin therapy may associate with decreased risk of macrovascular complications such as atherosclerosis and death in patients with type 2 diabetes, especially in those with high cardiovascular risk.

However, the possibility of excessive insulinization during insulin therapy has raised concerns about the potential risk for accelerated atherosclerosis and its complications. Recently, some studies demonstrated the increased risk of atherosclerotic lesions and its complications in T2DM patients receiving insulin treatment. For example, Colayco et al. in the Kaiser Permanente T2D patient registry cohort found that an increase in length of insulin exposure was associated with about a 2.5-fold increase in hazard of CV events (25). Additionally, a case-control study of 836 T2DM patients reported higher cardiovascular disease risk in subjects with high-dose insulin therapy (6). Furthermore, Kronmal et al. found that fasting serum insulin levels were associated with increased incident coronary heart disease in insulin-treated subjects (26). Likewise, Gamble et al. also observed a consistently increased risk of mortality associated with increased insulin exposure (8). Compared with the subjects without insulin exposure, there was an approximate 180% increased risk of all-cause mortality in those with high levels of insulin exposure in type 2 diabetes (8).

Moreover, according to the results of the Action to Control Cardiovascular Risk in Diabetes (ACCORD) trial, the risk of CV death was higher in the intensified treatment group than in the conventional treatment group, which may be explained by the use of insulin in the intensified treatment group at a higher rate (79 vs. 62%, *p* < 0.001) and dose (0.41 vs. 0.30 units/kg/day, *p* < 0.001) than in the standard treatment group (27). However, a recent study showed that insulin dose was not associated with increased mortality in the ACCORD study (28). Our present study also found that the patients receiving insulin therapy had significantly higher prevalence of carotid plaque even adjusting for traditional cardiovascular factors such as hypertension and dyslipidemia compared with the patients without receiving insulin therapy, which was consistent with a recent study by Herman et al. (29). Herman et al. found that insulin therapy was positively and independently associated with increased risk of macrovascular complications in T2DM (29). However, another cohort study, the ORIGIN trial, found that the early use of basal insulin targeting normal fasting plasma glucose levels neither reduced nor increased cardiovascular outcomes as compared with guideline-suggested glycemic control (30). Since this study included a population of early-stage diabetic patients, it may differ from our findings.

In the present study, the patients receiving insulin treatment accompanied more severe cardiovascular risk factors such as higher LDL-C and blood pressure levels, which may be one of the causes that insulin-treated patients suffered from a higher risk of carotid plaque. For example, we found that the patients receiving insulin therapy had higher levels of TC and LDL-C, which are among the most important risk factors for atherosclerosis (31). Consistent with our findings, several studies also indicated that the increased atherosclerosis risk in T2DM subjects was likely due to the myriad of metabolic abnormalities such as dyslipidemia, oxidized and glycolated proteins, and elevation of inflammatory cytokines caused by insulin resistance and hyperglycemia (32–34).

More importantly, insulin resistance may be the key cause leading to increased risk of carotid atherosclerosis in insulin-treated T2DM patients in the present study. Interestingly, although islet function reflected by HOMA2- B% was significantly poor in T2DM patients receiving insulin treatment compared with those without receiving insulin treatment, insulin levels and HOMA-IR were obviously higher in the patients receiving insulin therapy than in those without receiving insulin therapy, which indicated the more severe insulin resistance in insulin-treated T2DM patients.

Presently, it is well established that the insulin resistance is closely associated with increased risk of atherosclerosis and its complications in both normal and abnormal glucose tolerance subjects. Insulin resistance and the consequent hyperinsulinemia are strongly associated with central obesity, hypertension, and dyslipidemia, in which all factors contribute substantially to CV risk. Hyperinsulinemia has also been shown to account for risk of atherogenesis through promoting arterial smooth muscle cell proliferation and pro-inflammatory molecules formation (35). Both *in vivo* and *in vitro* studies have demonstrated that insulin can promote atherogenesis (36, 37). For example, insulin could lead to the accumulation of lipids in vascular wall with subsequent acceleration of inflammation and extracellular matrix remodeling, which are key features of atherosclerosis development (37).

On the other hand, multiple prospective studies have linked insulin resistance to accelerated atherosclerosis in patients with type 2 diabetes (38–40). For example, the Verona Diabetes Complications Study found that baseline CVD risk increased by 31% per each unit increase in log (HOMA-IR) in T2DM patients when controlling for multiple cardiovascular risk factors after 4.5 years of follow-up, in which insulin resistance was assessed by HOMA of insulin resistance (HOMA-IR) (38). Moreover, individuals in the highest quintile of insulin resistance still had a 1.48-fold increased incidence of CVD (38). Likewise, in the San Antonio Heart Study, after adjustment for multiple covariates, insulin resistance indicated by HOMA-IR was also significantly associated with increased risk of CVD (39). Additionally, a strong relationship between HOMA-IR and carotid intimal media thickness has also been demonstrated in the Brazilian Longitudinal Study of Adult Health study (41). Presently, the results of CIMT and carotid plaque in evaluating cardiovascular risk vary among different studies (42–44). For example, a meta-analysis including 119 randomized controlled trials showed that each 10 μm/year reduction in CIMT was associated with a relative risk reduction in CVD (43). Contrarily, another meta-analysis showed that carotid plaque is a more accurate predictor of cardiovascular events than CIMT (44). Therefore, insulin resistance is significantly associated with increased atherosclerosis risk in T2DM patients, which partly explains the increased risk of carotid plaque in our study.

Our study has potential clinical significance, because it may be beneficial for the use of medications such as metformin and GLP-1 receptor agonists in T2DM patients receiving insulin therapy, which can be regarded as insulin sensitisers and insulin sparing drugs to reduce the risk of atherosclerotic lesions and its complications. For example, the United Kingdom Prospective Diabetes Study (UKPDS) found that metformin is an effective drug for insulin resistance and the only treatment that successfully reduces the incidence of myocardial infarction (1). In addition, the use of GLP-1 receptor agonists in T2DM have also been associated with improvement in cardiovascular risk (45). Moreover, SGLT-2 inhibitors therapy improves insulin sensitivity and ameliorates insulin resistance. The addition of SGLT-2 inhibitors has also been proved to have cardiovascular benefit (46). A recent meta-analysis showed that SGLT-2 inhibitors reduced the risk of cardiovascular death, nonfatal myocardial infarction, and nonfatal stroke by 16% for the primary endpoint. There are limitations to our study. First, since this was a cross-sectional study, thecausal association between insulin therapy and carotid atherosclerotic lesions in type 2 diabetes cannot be verified and needs further clarification in future prospective studies. Second, even though we controlled for multiple confounding factors, variables not included in the analysis may affect the results of the present study. However, we adjusted as many variables as possible in our analysis. Third, as this was a single-centre study, multi-centre studies are needed to further explore the relationship between insulin treatment and atherosclerosis in type 2 diabetes. Fourth, the subjects in the present study were hospitalized patients who were more likely to be treated with insulin because of poor blood glucose control, which may not fully reflect the characteristics of the entire diabetic population. Fifth, there is no consensus on the effect of duration of insulin administration on the incidence of atherosclerotic lesions in current studies. Therefore, the 6 months' duration of insulin therapy maybe not enough to observe the effects of insulin treatment on atherosclerotic lesions.

In conclusions, insulin treatment in type 2 diabetes was closely associated with increased risk of carotid atherosclerotic lesions, which partly attributed to severe insulin resistance in patients receiving insulin therapy. The use of medications reducing insulin resistance such as metformin may be beneficial in T2DM patients receiving insulin treatment.

## Data Availability Statement

The original contributions presented in the study are included in the article/Supplementary Material, further inquiries can be directed to the corresponding author/s.

## Ethics Statement

The studies involving human participants were reviewed and approved by the ethics committee of Shanghai Jiao Tong University Affiliated Sixth People's Hospital. The patients/participants provided their written informed consent to participate in this study.

## Author Contributions

All authors listed have made a substantial, direct and intellectual contribution to the work, and approved it for publication.

## Conflict of Interest

The authors declare that the research was conducted in the absence of any commercial or financial relationships that could be construed as a potential conflict of interest.
